# Idiopathic Ventricular Tachycardia Arising From the Right Ventricular Apex

**DOI:** 10.1016/s0972-6292(16)30608-8

**Published:** 2013-03-07

**Authors:** Konstantinos P Letsas, Michael Efremidis, Spyros Tsikrikas, Antonios Sideris

**Affiliations:** Second Department of Cardiology, Laboratory of Invasive Cardiac Electrophysiology, Evangelismos General Hospital of Athens, 10676, Athens, Greece

**Keywords:** idiopathic ventricular tachycardia, right ventricular apex, catheter ablation

## Abstract

We present a rare case of idiopathic ventricular tachycardia arising from the right ventricular apex. The electrocardiographic and electrophysiological characteristics of this tachycardia are discussed.

## Case Presentation

**Figure 1:**
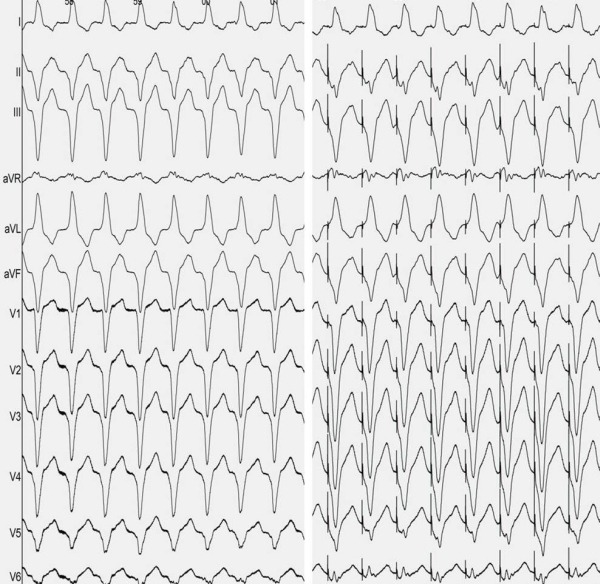
a. ECG tracing during ventricular tachycardia; b. Pace-mapping at right ventricular apex demonstrating near 'perfect match' (11 of 12 leads) with the morphology of the tachycardia.

A 67-year-old male presented to the emergency department complaining of dizziness. ECG on admission showed sustained monomorphic ventricular tachycardia (VT) with LBBB morphology, left superior axis and negative QRS concordance in precordial leads (Figure 1a). Electrical cardioversion restored sinus rhythm. Baseline ECG during sinus rhythm was unremarkable. Family history was negative for sudden cardiac death. Frequent premature ventricular contractions (PVCs) with the same QRS morphology were also recorded. Transthoracic echocardiography and coronary angiography ruled out structural heart disease. Cardiac magnetic resonance imaging (MRI) did not reveal any right ventricular fatty infiltration suggestive of arrhythmogenic ventricular cardiomyopathy/dysplasia (ARVC/D).

**Figure 2:**
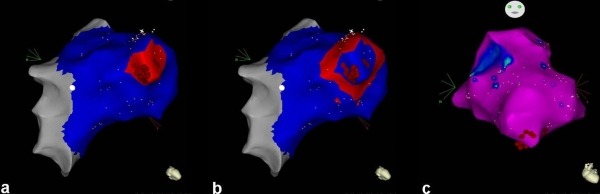
a and b. Propagation map showing a radial spread of activation arising from a discrete region of the right ventricular apex; c. Right ventricular voltage mapping showing the absence of any low voltage areas suggestive of scar.

After obtaining an informed consent, an electrophysiological study was carried out, where a detailed voltage and activation map was performed using a 3-dimentional electroanatomical mapping system (CARTO 3, Biosense Webster, Inc., Diamond Bar, CA, USA). Tachycardia was easily reproduced by programmed right ventricular stimulation, particularly after isoproterenol infusion. Left ventricular mapping was initially performed using a 4-mm non-irrigated tip deflectable catheter (NAVISTAR, Biosense Webster). Activation mapping failed to reveal any areas in the left ventricle with early local activation times. Therefore, right ventricular mapping was subsequently performed. The earliest site of ventricular activation (preceding the onset of the surface QRS by 34 ms) was identified at the apex of the right ventricle. Pace-mapping at this location demonstrated a good match (10 of 12 leads) with the morphology of the VT (Figure 1b). Propagation map revealed a radial spread of activation from a discrete region of the right ventricular apex (Figure 2a and b). Right ventricular voltage mapping failed to show any low voltage areas suggestive of scar (<1.5mV) (Figure 2c). The first radiofrequency (RF) energy application (target temperature of 60 ºC, 30 W, 60 s) abolished all PVCs. Additional RF applications were delivered at this region in order to ensure a long-term success. Programmed right ventricular stimulation, with and without ispoproterenol, failed to induce the tachycardia. Six months after the procedure, the patient is free from arrhythmias.

## Discussion

Idiopathic right ventricular VTs/PVCs usually display an outflow tract or annular (tricuspid) origin [1]. Tada et al. have reported idiopathic VTs/PVCs arising from the tricuspid valve exhibit an LBBB morphology with left superior axis and a transition zone beyond V3 [2]. Idiopathic VTs/PVCs arising from the right ventricular apex has been rarely described [3,4]. Navarrete has reported the first case of idiopathic right ventricular apical VT [3]. In this case, an LBBB morphology with left superior axis and a late precordial transition zone (V5-V6) during the tachycardia was described [3]. In a recent study, Van Herendael et al. have shown that idiopathic right ventricular apical VTs/PVCs display a late precordial transition zone (≥V6), negative QRS complexes in all inferior leads, and smaller R wave in lead II and S wave in lead aVR compared to VTs/PVCs arising from the basal segments or the tricuspid valve [4].

In our case, VT displayed a negative QRS concordance in precordial leads, which is not suggestive for the tachycardia origin. Therefore, both left and right ventricular activation mapping was performed. Structural heart disease including ARVC/D was excluded by transthoracic echocardiography, coronary angiography and cardiac MRI, while right ventricular voltage mapping failed to show any scar regions. A focal mechanism was suggested, possibly through triggered activity, since programmed right ventricular stimulation under isoproterenol infusion could easily reproduce the arrhythmia. However, an exercise stress test that could add more data regarding the arrhythmia mechanism was not performed. Right ventricular papillary muscles and/or a moderator band have been proposed as sources of triggers for ventricular arrhythmias [2,5]. A Purkinje potential at the successful site of ablation at the base of the papillary muscle has been identified in some patients, suggesting that the Purkinje fibers might be involved in the arrhythmogenic substrate [5]. However, the right ventricular Purkinje network is usually implicated in the genesis of polymorphic ventricular tachycardia or ventricular fibrillation [6,7].
